# Emerging Role of Extracellular Vesicles as Biomarkers in Neurodegenerative Diseases and Their Clinical and Therapeutic Potential in Central Nervous System Pathologies

**DOI:** 10.3390/ijms251810068

**Published:** 2024-09-19

**Authors:** Michele Malaguarnera, Andrea Cabrera-Pastor

**Affiliations:** 1 Departamento de Psicobiología, Facultad de Psicología y Logopedia, Universitat de València, 46010 Valencia, Spain; michele.malaguarnera@uv.es; 2 Departamento de Enfermería, Facultad de Enfermería y Podología, Universitat de València, 46010 Valencia, Spain; 3 Departamento de Farmacología, Facultad de Medicina y Odontología, Universitat de València, 46010 Valencia, Spain; 4 Fundación de Investigación del Hospital Clínico Universitario de Valencia, INCLIVA, 46010 Valencia, Spain

**Keywords:** extracellular vesicles, exosomes, biomarker, therapy, MSC, clinical trial, CNS diseases

## Abstract

The emerging role of extracellular vesicles (EVs) in central nervous system (CNS) diseases is gaining significant interest, particularly their applications as diagnostic biomarkers and therapeutic agents. EVs are involved in intercellular communication and are secreted by all cell types. They contain specific markers and a diverse cargo such as proteins, lipids, and nucleic acids, reflecting the physiological and pathological state of their originating cells. Their reduced immunogenicity and ability to cross the blood–brain barrier make them promising candidates for both biomarkers and therapeutic agents. In the context of CNS diseases, EVs have shown promise as biomarkers isolable from different body fluids, providing a non-invasive method for diagnosing CNS diseases and monitoring disease progression. This makes them useful for the early detection and monitoring of diseases such as Alzheimer’s, Parkinson’s, and amyotrophic lateral sclerosis, where specific alterations in EVs content can be detected. Additionally, EVs derived from stem cells show potential in promoting tissue regeneration and repairing damaged tissues. An evaluation has been conducted on the current clinical trials studying EVs for CNS diseases, focusing on their application, treatment protocols, and obtained results. This review aims to explore the potential of EVs as diagnostic markers and therapeutic carriers for CNS diseases, highlighting their significant advantages and ongoing clinical trials evaluating their efficacy.

## 1. Introduction

In recent years, there has been a significant increase in research focusing on the development of clinical applications using extracellular vesicles (EVs). These vesicles are gaining attention for their potential roles as both diagnostic markers and vehicles for therapeutic delivery [1,2]. It has led to a significant increase in the number of clinical trials involving EVs, with studies targeting diverse disease areas such as neurodegenerative diseases, cancer, inflammation, and immunology [3,4,5].

EVs are particles released from cells, delimited by a lipid bilayer, and unable to replicate on their own. EVs can be classified into different types based on their origin, such as exosomes and ectosomes. Exosomes refer to EVs from internal compartments of the cell that are released via the multivesicular body, while ectosomes (also known as microvesicle, microparticle) refer to EVs from the cell surface. However, most EV separation techniques do not enrich for EVs produced by different mechanisms, and definitive characterization of biogenesis-based subtypes is also difficult as there are no universal molecular markers of ectosomes, exosomes, or other EV subtypes. For this reason, it is advisable to use the term EVs to describe the diverse population of EVs more accurately [6]. Moreover, there is a growing awareness of a wide diversity of non-vesicular extracellular particles (NVEPs), which are multimolecular assemblies released from cells that lack a lipid bilayer, and these often co-separate with EVs. Therefore, when EVs and NVEPs cannot be fully distinguished from each other, MISEV 2023 proposes using the nomenclature “extracellular particle” (EP) or the terms “EV preparation” or “EV-containing preparation” as an umbrella term for all particles outside the cell, including both EVs and NVEPs [6].

The content of EVs is quite heterogeneous. According to the MISEV2023, EVs include transmembrane (or GPI-anchored) proteins associated with the plasma membrane and/or endosomes, such as the tetraspanin family (CD9, CD63, and CD81), Major Histocompatibility Complex Class I or II, integrins (ITGA*/ITGB*), transferrin receptor (TFR2), LAMP1/2, heparan sulfate proteoglycans including syndecans (SDC), EMMPRIN (BSG), ADAM10, glypicans, 5′-nucleotidase CD73 (NT5E), complement-binding protein, and CD59. While their content may vary, there is consensus on the presence of heat shock proteins (HSP70 and HSP90), actin, flotillins, proteins from the endosomal sorting complex required for transport (Alix and TSG101), caveolins, and syntenin [6,7]. Their cargoes include DNA, mRNA, miRNA, non-coding RNA, lipids, and metabolites, as well as cytoplasmic and membrane proteins involved in regulating intercellular signaling in both physiological and pathophysiological processes [8,9,10]. Furthermore, EVs have been shown to be involved in the transport of cellular waste as well as nutrients. They can also modulate extracellular matrix properties, contribute to mineral formation in bone tissues, and play a role in ectopic calcification [11,12]. The components of EVs, such as specific proteins, lipids, and nucleic acids, serve as molecular signatures that reflect their cellular origin. These molecular markers not only provide insights into the source cells but also influence the behavior of EVs, including their uptake by target cells, their biodistribution, and their interaction with the immune system. For example, tetraspanins like CD9 and CD63 are associated with the ability of EVs to facilitate intercellular communication and modulate the immune response [13]. Additionally, the presence of certain integrins on EVs can direct them to specific tissues, suggesting that EVs may play a role in tissue repair or pathological processes such as cancer metastasis [14]. Moreover, the cargo within EVs, including RNA and proteins, can alter gene expression and cellular function in recipient cells, highlighting their potential roles in disease progression and therapy. Their reduced immunogenicity and their ability to cross the blood–brain barrier make them an attractive and innovative option as biomarkers and therapeutic agents [15,16]. In fact, EVs, found in various body fluids, have the potential to serve as excellent biomarkers, especially for the early detection of diseases.

The role of EVs in central nervous system (CNS) disorders has gathered growing interest, leading to extensive research into their biological origins, material composition, transport mechanisms, intercellular signaling, and distribution in body fluids. In CNS disorders, they are currently employed for diagnosis, and their application as a drug delivery system is viewed as a promising treatment tool [17]. EVs, when used for therapeutic purposes, offer distinct advantages. Generally, due to their cellular origin, EVs exhibit innate biocompatibility and biodegradability. This biocompatibility arises from their composition, which mirrors that of the cells from which they are derived, including lipids, proteins, and nucleic acids. This similarity allows EVs to interact with biological systems without eliciting a significant immune response [18]. This intrinsic property minimizes the risk of immune rejection or adverse reactions, presenting them with a more secure alternative to synthetic carriers for drug delivery [19,20]. Furthermore, the presence of the lipid bilayer structure allows for the efficient encapsulation of cargo and their delivery to targeted sites [21]. Furthermore, EVs can be engineered to contain specific targeting molecules on their surface, facilitating selective delivery of therapeutic cargo to desired cells or tissues [22]. EVs derived from stem cells have been shown to have beneficial effects in promoting tissue regeneration, modulating the immune system, promoting angiogenesis, and repairing damaged tissues in a variety of conditions, including neurodegenerative diseases [23,24,25,26,27]. The aim of this review is to explore the potential of EVs to serve as both diagnostic markers and therapeutic carriers for CNS diseases.

## 2. Extracellular Vesicles as Biomarkers for CNS Diseases

EVs play a crucial role in the pathophysiology of CNS diseases. The alterations associated with CNS diseases induce changes in the content of EVs released by relevant cells, such as neurons and glia [28]. Given that EVs released by cells can cross the blood–brain barrier (BBB) [16], their isolation from peripheral blood facilitates detection and analysis using techniques such as chromatography size exclusion, Western blotting, enzyme-linked immunosorbent assay, flow cytometry, quantitative real-time polymerase chain reaction, or nanoparticle tracking analysis [29]. EVs are enclosed within a lipid bilayer, which shields them from enzymatic degradation, and this protection renders the content of EVs more stable compared to more vulnerable circulating DNA or RNA [30,31]. This has made EVs emerge as an attractive liquid biopsy approach for the diagnosis and prognosis of neurological disorders, offering real-time insights for early diagnosis and monitoring disease progression by serving as potential sensitive biomarkers that swiftly respond to CNS diseases [32,33,34].

### 2.1. EVs as Biomarker in Alzheimer Disease

Alzheimer’s disease (AD) is a neurodegenerative disorder characterized by amyloid-beta (Aβ) plaques and neurofibrillary tangles accumulation, leading to cognitive impairment [35]. Biomarkers may play a crucial role in early diagnosis, with molecular neuroimaging and fluid biomarkers confirming AD pathology [36]. Some of the markers have been found in EVs and, in a recent meta-analysis, have demonstrated a good diagnostic potential [37]. These key biomarkers include Aβ 40 and Aβ 42, and markers of AD-related metabolic disorders, T-tau (total) [38], and different phosphorylated forms of P-tau like phosphorylated at Thr-181 [39,40] and at Ser-396 [41,42].

Several authors have explored the mechanism by which EVs, containing amyloid precursor protein (APP) and its derivatives, disseminate to target cells. EVs serve as vehicles, transmitting neurotoxic forms of APP and tau between neuronal cells. These cargoes can be transferred directly to recipient cells or indirectly through the bloodstream or cerebrospinal fluid (CSF), ultimately leading to the accumulation of pathological AD proteins within receiving neuronal cells [43].

Furthermore, there is accumulating evidence indicating that the aggregation-prone proteins transfer between cells and propagate throughout the brain via EVs-mediated transport, contributing to disease onset and progression [40,44,45]. Moreover, it has been demonstrated that pathogenic proteins carried by EVs derived from brains with AD can traverse the BBB, suggesting its potential as bloodstream biomarker carriers for AD [46].

Analyzing the levels of various AD-related proteins in plasma EVs holds promise for identifying blood-based biomarkers at different stages of the disease. In fact, pathogenic proteins associated with AD were found to be more concentrated in plasma EVs compared to plasma. Therefore, plasma EVs should be considered as a reservoir of potential biomarkers because their use can enhance the sensitivity of certain markers that may be diluted or rapidly degraded in plasma [46].

In mice with AD, it has been shown that tau proteins in EVs originating from the CNS, identified by a potential CNS-specific marker known as L1 cell adhesion molecule (L1CAM), have been observed to be easily transported from the brain to the peripheral blood [47].

During mild cognitive impairment and AD, the amyloid β42 (Aβ42) levels, Aβ42/Aβ40 ratio, tau, ratio of phosphorylated tau at position 181 (p-tau-181) to tau, and miR-384 levels were increased in plasma EVs and were correlated with those in CSF [48]. Additionally, elevated levels of p-tau-181 and the tau/Aβ42 ratio in plasma EVs were found to be negatively correlated with cognitive scores [46,49]. Furthermore, older individuals with cognitive decline less severe than mild cognitive impairment or dementia exhibited higher levels of tau, p-tau-181, and p-tau-231 in neuronal extracellular vesicles (nEVs) compared to cognitively stable individuals [50]. Levels of p-tau-181 and Aβ42 in plasma neuron-derived EVs varied significantly with increasing age in patients with AD compared to controls, while levels of p-tau-S396 remained unchanged [42,51].

The differences in Aβ42, tau, p-tau-181, and p-tau-S396 levels in plasma neuron-derived EVs among control individuals, those with MCI, and those with AD were strongly correlated with CSF levels, and the diagnostic efficacy of these combined markers in EVs was comparable to that of CSF. Additionally, the combination of EVs biomarkers had greater diagnostic efficacy than each individual biomarker alone, and the peripheral blood nEVs biomarkers exhibited similar diagnostic performance as the CSF biomarkers [52,53]. Moreover, the combination of Aβ42 levels in plasma neuron-derived EVs along with a test assessing olfactory function, which appears to be altered across various neurodegenerative diseases, showed improved prediction of the conversion from MCI to AD dementia when used together [54].

These studies have shown that AD progresses from asymptomatic stages though MCI to moderate and severe dementia stages, which are associated with specific biomarkers, including elevated levels of EVs containing tau, p-tau-181, and p-tau-231. During the MCI stage, increased levels of Aβ42 and tau, as well as elevated p-tau-181/tau, Aβ42/Aβ40, and tau/Aβ42 ratios, are observed. Furthermore, these pathogenic AD proteins identified in plasma neuronal EVs have been demonstrated to be more reliable biomarkers for AD when used in combination rather than relying on individual markers alone.

Additionally, other biomarkers related to Aβ peptide and implicated in the advancement of AD pathology were found in EVs. For example, levels of two Aβ-binding proteins, alpha-1-antichymotrypsin (AACT), were elevated, while the level of C4b-binding protein alpha chain (C4BPα) was reduced in plasma EVs from AD. Consequently, these proteins were identified as promising candidates for EVs biomarkers in AD diagnosis [55].

Gelsolin, a protein involved in inhibiting Aβ fibril formation, were found to be lower in serum EVs of dementia patients compared to controls [56]. Conversely, the level of BACE1-antisense transcript (BACE1-AS) was significantly elevated in plasma EVs of patients with AD [57].

It has been shown that the components responsible for generating Aβ42 were found in two distinct groups of EVs derived from astrocytes (ADEs) and neurons (NDEs) in the plasma of both patients with AD and controls. Levels of key proteins, including BACE-1, γ-secretase, soluble Aβ42, soluble sAPPβ and sAPPα, and p-tau-181 and p-tau-S396, were significantly higher in ADEs compared to NDEs in both patients with AD and healthy individuals. This suggests that the cargo proteins within ADEs may offer valuable insights into cellular interactions and biomarkers in AD [58].

Additionally, increased levels of tau were detected in EVs derived from microglia in patients with AD compared to controls [59]. These results suggest a potential role of microglia-derived EVs in tau propagation within the human brain and the progression of AD pathology. In a mouse model of AD, there has been described consistent levels of Aβ and tau in plasma neuronal EVs and astrocyte-derived EVs with their levels in the brain [60].

In addition to the biomarkers Aβ42 and p-tau181, the protein neurofilament light chain (NfL), predominantly found in nerve cells, has been proposed as a potential biomarker for several neurological conditions, including AD. Following axonal injury and neurodegeneration, NfL is released into the extracellular space. A study comparing patients with AD to healthy controls showed higher levels of NfL patients with AD EVs from plasma [61]. A proteomic analysis revealed ten proteins as potential EVs biomarkers from plasma in patients with sporadic AD, and these proteins included distinct immunoglobulins, fibronectin, and apolipoproteins [62].

Not only could proteins in EVs be interesting biomarkers for AD but also lipids and nucleic acids such as miRNAs. A recent study showed differences in selected lipid species of glycerolipids, glycerophospholipids, lysophospholipids, and sphingolipids across three distinct brain-derived EV subpopulations from plasma samples, after removal of lipoproteins, from individuals with AD and healthy controls [63].

The diagnostic value of miRNAs from plasma/serum-derived EVs has also been assessed, and several miRNAs have been proposed as potential biomarkers. Serum-derived EVs miR-135a and miR-384 were upregulated, and miR-193b was downregulated in MCI and AD, indicating their potential as biomarkers for the early diagnosis of AD [64]. miRNAs-125b-3p and miRNA-451a are differentially expressed in AD compared to the healthy group, with high sensitivity and specificity [65]. Enrichment analysis of their target genes showed that they are related to neuroactive ligand–receptor interactions, the PI3K-Akt signaling pathway, the Hippo signaling pathway, nervous system-related pathways, cytokine–cytokine receptor interactions, and the PI3K-Akt signaling pathway [65]. miR-483-5p and miR-502-5p from plasma EVs are at high levels in elderly people with MCI compared to controls, suggesting their potential as promising noninvasive biomarkers [66].

Moreover, apart from EVs containing pathogenic proteins, lipids or miRNAs obtained from CSF and plasma, several studies have highlighted the potential use of urinary or salivary EVs as potential biomarkers in the early stages of AD. Urinary EVs from patients with AD exhibited significantly higher levels of AD pathological proteins compared to healthy controls. Additionally, the amount of urinary EVs was higher in patients with AD than in healthy subjects [67]. Similarly, salivary EVs from AD and cognitive impaired patients showed markedly increased expression levels of Aβ oligomer/fibril, Aβ, and phosphorylated tau compared to healthy subjects, providing insights into the pathological progression of AD [68]. A recent study showed that the miRNA-485-3p concentration in salivary-derived EVs isolated from patients with AD was positively correlated with Aβ deposition in the brain and demonstrated high predictive value for Aβ-PET positivity [69].

A summary of potential biomarkers in EV-containing preparations for AD, including the biofluid from which the EVs were isolated, the isolation technique used, the method of characterization, and their role as potential biomarkers, is shown in Table 1.

### 2.2. EVs as Biomarker in Parkinson Disease

The necessity of identifying new biomarkers for Parkinson’s disease (PD) is driven by the fact that clinical symptoms, such as motor function alterations, appear decades after the neurodegeneration begins. Autolysosomal proteins such as cathepsin D, LAMP, or α-synuclein are suggested as diagnostic markers for early-stage PD [70,71,72,73,74]. Changes in the levels of plasma EV synaptic proteins, namely, SNAP-25, GAP-43, and synaptotagmin-1, are associated with motor decline in PD [75]. Elevated baseline plasma EV synaptic protein levels can predict increased deterioration of motor function, particularly postural instability and gait disturbance symptoms, in PD [75]. A follow-up study with patients with PD found significant differences in plasma EVs levels of α-synuclein, tau, and Aβ 1-42 compared to healthy controls. Elevated levels of tau alone or all three plasma EV proteins were predictive of motor and cognitive dysfunction progression at the 1-year follow-up [76]. These findings suggest that plasma EV α-synuclein, tau, and Aβ 1-42 are associated with brain pathology in PD.

A link has also been shown between alterations in plasma EV-derived cytokine levels and the progression of motor and cognitive symptoms in PD [77]. Baseline plasma EV-derived cytokine levels were correlated with clinical outcomes, with elevated levels of plasma EV-derived IL-1β and IL-6 identified as predictors of the progression of postural instability and gait disturbance [77]. These findings suggest that plasma EV-derived cytokines could serve as effective biomarkers for PD progression, highlighting the critical role of inflammation in the pathogenesis of the disease.

Neuronal EVs are emerging as a novel reservoir of biomarkers for neurodegenerative disorders. In fact, neuronal EVs α-synuclein, identified as a major protein associated with PD, has been reported higher in patients with early and advanced forms of the disease compared to healthy individuals [70,78,79]. The elevated release of α-synuclein in serum neuronal EVs precedes PD diagnosis, persists throughout disease progression, and correlates with protein aggregation, thereby enabling the prediction and differentiation of PD from atypical Parkinson’s syndromes [80,81]. Conversely, no significant difference in total α-synuclein concentration between patients with early and advanced PD was observed, underscoring the EVs as a more sensitive tool for diagnosis and severity assessment [82]. Moreover, the levels of α-synuclein, phosphorylated Tyr-181 tau, and phosphorylated insulin receptor substrate-1 in neuronal L1CAM-immunocaptured EVs extracted from plasma of PD patients emerge as valuable biomarkers for predicting cognitive outcomes [83]. The higher levels of ptau181 in the neuronal EVs of patients with PD also indicates that AD-related proteins are also responsible for cognitive impairment in PD [83]. It has also been demonstrated that pathological α-synuclein aggregation increases the release of EVs from astrocytes, partly due to α-synuclein-induced lysosomal dysfunction [84]. Furthermore, when isolating EVs enriched with astrocyte markers from plasma, higher levels of these EVs carrying total α-synuclein and α-synuclein aggregates were found in the PD group compared to healthy controls [84]. These findings show the potential of CNS-derived blood EVs as diagnostic, prognostic, and progression markers for neurodegenerative conditions. 

EVs isolated from serum, CSF, and brain lysates of patients with PD have been demonstrated to contain aggregated α-synuclein [85]. These vesicles can transfer the aggregated protein to healthy dopamine (DA) neurons, leading to the development of PD pathology both in vitro and in vivo [86,87,88,89,90,91,92]. 

A proteomic study found more differentially expressed proteins in CSF-derived EV samples compared to non-purified CSF, with minimal overlap between datasets [93]. This study suggests that CSF-derived EV samples may be more useful for biomarker discovery, particularly for neurodegeneration or general disease states. Protein biomarker candidates that differentiated healthy controls from PD in CSF-derived EVs were RALB, POR, SMPD3, MAPT, DDX6, ILK, OGN, CTSS, PPP1CC, FLII, IGKV1-8, SHMT1, and RPL35A [93].

Serum EVs miR-19b, miR-195, and miR-24 exhibit dysregulated expression patterns in PD and show promise as potential diagnostic biomarkers [94]. A recent study developed a molecular beacon-based assay to evaluate microRNA-containing EVs in plasma and showed that miR-44438-containing EVs could be a potential biomarker for the early detection and progress monitoring of α-synucleinopathies [95]. Other authors observed an opposite expression profile of miR-23b-3p in PD compared to age-matched healthy controls in plasma and plasma-derived EV fractions, where the expression of miR-23b-3p was increased in PD plasma while decreased in plasma-derived sEV fractions [96]. Reduced expression of miR-128 in EVs from PD patients’ plasma is involved in neural degeneration and in 6-OHDA-mediated neuronal apoptosis [97]. EVs derived from neurons and isolated from serum showed that a panel of 29 small RNAs is expressed differentially between patients with PD and controls, suggesting their potential utility as biomarkers for PD [98]. Other authors have shown that patients with REM sleep behavior disorder and higher levels of plasma EV-derived miR-7-5p, miR-4665-5p, miR-5001-3p, and miR-550b-3p are more likely to develop PD [99].

CSF EVs RNA molecules, including miR-153, miR-409-3p, miR-10a-5p, let-7g-3p, miR-1, and miR-19b-3p, has emerged as a potential biomarker with moderate robustness in distinguishing PD from both healthy individuals and those with other neurodegenerative conditions like Alzheimer’s disease [100].

Furthermore, EVs isolated from others biological fluids such as saliva and urine have been utilized for diagnostic purposes. EVs present in the saliva and urine of patients with PD may also harbor early biomarkers such as RNAs and miRNAs, which have the capability to differentiate between sporadic PD and specific inherited forms associated with DJ-1 and *LRRK2* mutations [101,102,103,104,105]. LRRK2 is involved in the trans-phosphorylation of Rab proteins [106]. Consequently, the level of Rab phosphorylation in EVs could help predict the PD stage. Indeed, increased phosphorylation of Rab10 was observed in the urine EVs of patients with PD with *LRRK2* mutations compared to controls, and it further correlated with the patients’ motor and cognitive impairment scores [107]. Other authors showed that PD was associated with increased Rab8 levels and decreased phosphorylation at pS910-LRRK2 and pS935-LRRK2 in urine EVs [108]. Therefore, PD EVs could not only help predict the disease but could also help identify molecular signatures linked to disease symptoms.

Moreover, abnormal expression of calbindin and SNAP23 in urinary EVs has been identified as another potential diagnostic biomarker for PD [103].

Although it remains unclear whether the levels of EV-derived biomarkers increase as PD advances and disease severity worsens, their diagnostic potential remains undeniable.

A summary of potential biomarkers in EV-containing preparations for PD, including the biofluid from which the EVs were isolated, the isolation technique used, the method of characterization, and their role as potential biomarkers, is shown in Table 2.

### 2.3. EVs as Biomarker in Amyotrophic Lateral Sclerosis

Amyotrophic lateral sclerosis (ALS) still lacks an efficient treatment and biomarkers. To identify effective and accessible biomarkers for ALS, studies have investigated differentially expressed proteins in EVs from blood samples of patients with ALS compared to control groups. Among these proteins, coronin-1a (CORO1A) increase in EVs isolated from the plasma of patients with ALS compared to controls [109]. The role of CORO1A in ALS pathogenesis was discovered, potentially affecting the disease onset and progression by blocking autophagic flux. The level of CORO1A rises proportionally with disease progression in both the plasma of patients with ALS and the spinal cord of ALS mice. Given its significant impact on ALS pathogenesis, CORO1A could serve as a potential biomarker for ALS [109].

Approximately 20% of familial cases of ALS result from mutations in the *Cu/Zn superoxide dismutase* (*SOD*) gene [110]. Mutations in the *SOD1* gene cause misfolding of the SOD1 protein in vivo, culminating in the development of toxic aggregates [111]. Sproviero et al. reported that comparing levels of SOD1 and ALS-associated biomolecules in plasma EVs between patients with ALS and healthy controls has provided evidence supporting further exploration of ALS-related SOD1 levels in various EV types and suggesting its potential as a biomarker for ALS [112].

Other authors, in a longitudinal follow-up study, obtained plasma-derived EV samples from patients with ALS at baseline and at intervals of 1, 3, 6, and 12 months thereafter [113]. The ratio of neurofilament light chain (NFL) to phosphorylated neurofilament heavy chain (pNFH) and the ratio of TAR DNA-binding protein-43 (TDP-43) was assessed in EVs. TDP-43, a member of the heterogeneous nuclear ribonucleoprotein family, plays a role in RNA processing and can form insoluble aggregates in the brains of patients with ALS [114]. The results showed that the TDP-43 ratio in plasma-derived EVs was increased at the 3-month and 6-month follow-up periods. After categorizing patients into rapid and slow progression groups, plasma-derived EV showed significantly higher levels of NFL, but not of pNFH, in the rapid progression group at baseline and at the 3-month follow-up, indicating NFL in EVs as a potential biomarker for disease progression [113]. However, additional research is necessary to determine the diagnostic effectiveness of these proteins.

In addition to CORO1A, SOD1, and TDP-43, other ALS-related targets are also contained in secreted EVs, although with a lower concentration. ALS-related mutations in FUS can lead to varying degrees of mis-localization of FUS in the cytoplasm, and the formation of stress granule-like structures. A prior study on familial ALS confirmed the presence of FUS in EVs, supporting the idea of FUS transmission between pathological cells, potentially aiding in ALS diagnosis [115]. Other ALS-related protein mutants found in EVs, such as valin-containing protein, sequestosome 1, and Tank-binding kinase 1, could also serve as diagnostic markers for ALS [116,117,118]. Other authors identified the INHAT repressor (NIR) as a protein that showed increased levels in EVs-enriched fractions from CSF and confirmed that NIR expression was reduced in motor neurons of sporadic patients with ALS [119]. These results suggest that NIR could serve as a potential biomarker protein for ALS.

In addition to proteins, EV miRNAs have emerged as promising tools for enhancing ALS diagnosis, as certain miRNAs can influence the expression of proteins involved in ALS. Researchers have also explored ALS-associated miRNA profiles in EVs obtained from the CSF or peripheral blood of patients. One such miRNA, miR-146a-5p, implicated in regulating synaptic plasticity and inflammatory response by targeting synaptotagmin1 and neuroligin1, exhibits altered expression in EVs from the CSF of patients with ALS [120]. However, its diagnostic utility remains uncertain. Other authors identified differential expression of 22 miRNAs in EVs from the plasma of patients with ALS compared to controls, and miR-15a-5p and miR-193a-5p, with high sensitivity and specificity, demonstrated promising diagnostic potential for ALS [121].

Similarly, miRNA analysis of neuronal-derived EVs from the plasma of patients with ALS revealed 30 miRNAs showing differential regulation in ALS plasma compared to healthy control plasma [122]. These dysregulated miRNAs are associated with synaptic vesicle-related pathways, with four of them also showing dysregulation in the motor cortex tissues samples of patients with ALS [122]. Another study employing the same methodology identified a potential miRNA signature in neuronal-derived EVs from the plasma of patients with ALS, including miR-146a-5p, miR-199a-3p, miR-151a-3p, miR-151a-5p, and miR-199a-5p, which were up-regulated in patients with ALS compared to healthy controls. Additionally, three miRNAs (miR-4454, miR-10b-5p, and miR-29b-3p) were found to be down-regulated in ALS [123,124]. 

EVs play a role in ALS pathogenesis through the transfer and subsequent intracellular accumulation of pathological proteins such as TDP-43, SOD1, CORO1A, NIR, and FUS. Studies have reported dysregulation of protein and microRNA cargos of EVs in models of ALS and in patients; however, further studies are required to enhance the efficacy of EV-based biomarkers for clinical diagnosis.

A summary of potential biomarkers in EV-containing preparations for ALS, including the biofluid from which the EVs were isolated, the isolation technique used, the method of characterization, and their role as potential biomarkers, is shown in Table 3.

## 3. Extracellular Vesicles in Clinical Trials

In recent years, several clinical trials have started to explore the clinical significance of EVs as novel biomarkers for AD diagnosis or drug response. For instance, in 2017, the University Hospital in Lille initiated recruitment of participants to assess tau levels in EVs isolated from cerebrospinal fluid (ClinicalTrials.gov identifier: NCT03381482; see Table 4). Similarly, in 2019, the University of Oxford launched the MICAD study (ClinicalTrials.gov identifier: NCT04121208; see Table 4) to investigate the efficacy of the drug JNJ-40346527 in inhibiting the colony stimulating factor-1 receptor, which regulates microglial cell activity. It was supported by research indicating that reducing the numbers of microglial cells may help to slow the progression of AD [125]. The MICAD study aimed to assess changes in both protein biomarkers and the number of EVs as indicators of altered microglial cell activity in the brain, but the study closed after only two participants were recruited.

Other group from Bejjng, China, reported a retrospective study on the combination of blood neuro-EV proteins (GAP43, neurogranin, SNAP25, and synaptotagmin) as a predictor of the onset of AD 5 to 7 years in advance of the cognitive decline. Recently, they started a longitudinal study (ClinicalTrials.gov identifier: NCT05163626; see Table 4), at Xuanwu Hospital in Beijing in 2024. In this study, they are planning to assess the effects of a long-term combined aerobic exercise and cognitive training program on cognitive function, as well as the predictive biomarkers found in blood neuro-EV synaptic proteins, in seniors at increased risk of AD with abnormally decreased levels of these biomarkers. The primary objectives include determining the relationship between changes in biomarkers and cognitive function in this population, and confirming the predictive value of blood neuro-EV synaptic proteins for AD.

In the clinical trial NCT03275363 (Table 4), the University of Hong Kong Neurocognitive Disorder (NCD) Cohort is a hospital-based, prospective, observational study involving older Hong Kong Chinese adults with cognitive impairment. The study specifically focuses on patients with subjective cognitive decline and mild cognitive impairment, with particular emphasis on examining EV biomarkers isolated from the blood that predict cognitive and functional decline. Despite the study first being registered on ClinicalTrials.gov and listed as recruiting in 2017, no updates have been registered to date.

In another study, subjects with mild cognitive impairment were treated with curcumin and yoga with their respective placebo or non-treated group. This trial may also provide RNA sequencing on EVs to investigate their potential as clinical prognostic and diagnostic markers linked to AD development and the used treatments (ClinicalTrials.gov identifier: NCT01811381; see Table 4). Although this study was registered on ClinicalTrials.gov in 2013, the most recent update in 2020 indicates that the study is ongoing and still recruiting participants.

A recent study presented results from a randomized controlled trial (ClinicalTrials.gov identifier: NCT01681602; see Table 4) evaluating changes in the neuroprotective proteins proBDNF, BDNF, and humanin in plasma neuron-derived EVs from patients with mild-to-moderate AD [126]. This study suggests that exercise may improve cognitive function by increasing the neuroprotective factors in neuron-derived EVs. Patients with AD with the APOE ε4 variant appear to be more responsive to these benefits. The lack of change in specific exerkines after exercise suggests that different pathways might be involved.

In 2019, the National Institute of Aging in Baltimore initiated a proof of concept to examine the potential of empagliflozin, an antidiabetic drug, to enhance ketone levels and promote neuronal health, thereby potentially slowing down the onset and progression of cognitive decline (ClinicalTrials.gov identifier: NCT03852901; see Table 4). In this study, both total and neuronal-EVs were isolated from plasma to investigate whether increased ketone bodies could activate IGF-1 and insulin signaling pathways in non-diabetic individuals. They found that after the first dose, empagliflozin significantly elevated pIGF-1R, pIR, and downstream mediators of IGF and insulin signaling pathways (pY-IRS-1 and pAKT) in neuronal-EVs [127]. These studies provide a promising approach for identifying non-invasive diagnostic and prognostic biomarkers of neurodegeneration, and they offer new perspectives on clinical tools for tracking the progression of AD in comparison to other neurodegenerative conditions.

Currently, there are six clinical studies aimed at investigating the potential of EVs as biomarkers for PD. The first study was initiated at the University of Alabama at Birmingham (NCT01860118; Table 4) to explore biomarkers linked to PD susceptibility and/or progression in EVs proteomes obtained from patients with PD compared to controls. Additionally, the study aimed to determine whether LRRK2 expression and/or phosphorylation were significantly reduced in the EVs of individuals treated with the potent LRRK2 kinase inhibitor sunitinib (a multi-kinase inhibitor compound) in order to establish an assay for the on-target effects for future LRRK2 inhibitor clinical trials. They found that elevated levels of Ser(P)-1292 LRRK2 and higher levels of pT73-Rab10—a substrate of LRRK2 kinase—in EVs correlate with cognitive impairment, difficulty in daily activities, and worse disease progression in idiopathic PD [105,107,128]. A proteomic analysis of urinary EVs identified SNAP23 and calbindin as the most elevated proteins linked to neurological diseases in PD, achieving an 86% prediction success rate in the discovery cohort and 76% in the replication cohort, suggesting their potential as reliable biomarkers for PD diagnosis [103].

Two other studies, sponsored by the Michael J. Fox Foundation for Parkinson’s Research (NCT03775447, Fox BioNet Project Extracellular Vesicles ECV-003; and NCT04603326, Fox BioNet Project ECV-004), are focused on optimizing the isolation of EVs from human cerebrospinal fluid (CSF) to detect levels and activity of LRRK2, a protein associated with PD. These clinical trials do not yet show published results. The other three clinical trials, sponsored by Fondazione Don Carlo Gnocchi Onlus (ClinicalTrials.gov identifier: NCT05902065, NCT05452655, and NCT05320250), aim to assess novel serum biomarkers within neuronal-derived EVs for evaluating rehabilitative outcomes in patients with PD and to validate molecules isolated from either saliva or salivary EVs as new biomarkers for distinguishing between PD and atypical parkinsonism (Table 4). Some results from these studies showed that increased α-synuclein in neuron-derived EVs from serum can predict and differentiate PD from atypical parkinsonian syndromes, and that the evaluation of α-synuclein and aggregated tau in neuron-derived EVs enables us to distinguish between PD and atypical parkinsonian syndrome [81]. Also, they found a positive and direct correlation between NDEVs’ oligomeric α-Syn concentration and higher REM sleep behavior disorder (RBD) screening questionnaire scores in patients with PD, independent of age, disease duration, PD stages, and motor impairment severity [129]. A lower concentration of NDEVs syntaxyn-1, a SNARE complex component, was observed in RBD compared to non-RBD PD subjects [129].

RNA sequencing was performed to determine non-coding RNA changes in circulating EVs, which has also been studied as a biomarker for monitoring disease development in patients with traumatic brain injury (ClinicalTrials.gov identifier: NCT05279599; Table 4). Although the completion of this study was estimated for November 2022, the most recent update indicates that the study is ongoing and still recruiting participants.

In two clinical trials, researchers are exploring the use of EVs as biomarkers in stroke. In trial NCT05370105, a surface plasmon resonance imaging (SPRi)-based biosensor is being tested to detect and characterize blood EVs from stroke patients before and after rehabilitation, aiming to personalize therapies by identifying new biomarkers. In trial NCT06319742, the focus is on using EVs to diagnose and predict outcomes for transient ischemic attacks and strokes. This involves characterizing EVs through various techniques to differentiate between brain ischemia and stroke mimics, thereby enhancing patient management and therapy (Table 4).

EVs, characterized by their small size, low immunogenicity, and efficient delivery capability, can infiltrate brain endothelial cells via several pathways, including membrane fusion with target cells, receptor–ligand-interaction-mediated internalization, and endocytosis [130]. EVs with cargo traverse the BBB, a critical step for brain-targeted therapies, thereby modulating molecular mechanisms involved in CNS diseases. Also, EVs can act as nano-carriers for small molecules, particularly miRNA. To improve the targeting efficacy of EVs, they commonly undergo engineering modifications prior to administration, signifying promising research in the EV therapy for CNS disorders [131].

It is noteworthy that stem-cell-derived EVs have demonstrated antioxidant, anti-inflammatory, and antiapoptotic properties in various neurological, endocrine, and other organ-specific disorders [25,132].

Mesenchymal stem cell (MSC)-EVs from various sources, such as bone marrow, umbilical cord, and adipose tissue, have shown beneficial effects in neurological disorders. The potential therapeutic applications of MSC-EVs include the following: (a) enhancing neurogenesis and angiogenesis, reducing apoptosis; (b) promoting neurite remodeling and synapse growth [133]; and (c) reducing inflammatory response by suppressing microglia and inflammatory factors [134,135]. These beneficial outcomes are primarily attributed to MSC-EVs interacting with different types of recipient cells through the transfer of non-coding RNAs, particularly microRNAs, and bioactive proteins. 

A clinical trial was initiated in Ruijin Hospital, Shanghai, China (ClinicalTrials.gov Identifier: NCT04388982, Table 5) to assess the safety and effectiveness of exosomes derived from allogenic adipose mesenchymal stem cells (MSC-exos) in patients with AD. This phase I/II clinical trial investigates the impact of intranasal administration of different doses of MSC-exos over a 48-week period on individuals with mild to moderate AD-related dementia. Recent results published from this study showed that intranasal administration of allogenic human adipose MSC-exos was safe and well-tolerated, and a dose of at least 4 × 10^8^ particles could be selected for further clinical trials [136].

EVs derived from multipotent mesenchymal stromal cells (MSCs) promote neurovascular remodeling and functional recovery after stroke. Animal studies have shown that EVs treatment markedly increased the number of newly formed doublecortin (a marker of neuroblasts) and von Willebrand factor cells (a marker of endothelial cells). Intravenous administration of MSC-generated EVs post stroke improves functional recovery and enhances neurite remodeling, neurogenesis, and angiogenesis and represents a novel treatment for stroke. Additionally, some studies have shown that miR-124-loaded EVs ameliorate brain injury by promoting neurogenesis. Based on the background provided, the NCT03384433 trial aims to assess the improvement in patients with acute ischemic stroke who received MSC-derived EVs (Table 5). A recent study showed that intraparenchymal implantation of MSC-EVs in five ischemic stroke patients had no adverse effects after the intervention [137]. It is suggested that local injection of EVs following a cerebral artery infarct can be safe, and in the future, it could be used as a supportive, restorative, and preventive treatment for patients suffering from acute ischemic stroke and post-ischemic disability.

The NCT04202770 and NCT04202783 clinical trial aims to enhance the delivery of growth factors and anti-inflammatory agents to specific targets using focused transcranial ultrasound before intravenous infusion of EVs. EVs, which play a role in intercellular signaling, can naturally cross the blood–brain barrier when delivered intravenously. EVs from MSC have shown anti-inflammatory and pro-growth effects in preclinical models and clinical reports. Focused ultrasound, known to enhance local blood flow, is proposed as a non-invasive method to target therapeutic delivery. The NCT04202770 study uses focused ultrasound to enhance the delivery of intravenous EVs to the subgenual area for patients with refractory depression, the amygdala for patients with anxiety, and the hippocampus for patients with cognitive impairment due to neurodegenerative diseases.

The NCT05490173 study (Table 5) proposes a blinded randomized controlled trial to assess the impact of intranasal administration of EVs derived from MSCs on long-term neurodevelopmental outcomes in extremely low birth weight (ELBW) infants. EVs from MSCs have shown potential in various aspects of neuroprotection and health promotion in premature newborns. ELBW infants will be randomly assigned to receive either EVs or no treatment. The primary outcome measure includes evaluating the incidence of death, severe intraventricular hemorrhage, cystic periventricular leukomalacia, or other brain injuries, along with major neurodevelopmental impairments at 36 months of corrected age. Secondary outcomes involve short-term outcomes and safety analyses. By investigating these outcomes and underlying mechanisms, the study aims to enhance the quality of life for ELBW infants.

The NCT05886205 study (Table 5) addresses the need for effective treatments for refractory focal epilepsy as 30% of epilepsy patients do not respond to standard treatments, with most cases being focal epilepsy. Stem-cell-derived EVs contain bioactive substances that can inhibit apoptosis, reduce inflammation, promote angiogenesis, inhibit fibrosis, and enhance tissue repair. Induced pluripotent stem cells (iPSCs) originate from single-cell amplification, with infinite proliferation ability, providing a consistent and stable source for EVs. The NCT05886205 is a single-center, open-label clinical trial that aims to evaluate the safety, tolerability, and preliminary efficacy of iPSCs-derived EVs nasal drops in treating focal refractory epilepsy.

Following this line of research, NCT06138210 (Table 5) is a multicenter, randomized, double-blinded, placebo-controlled, dose-escalation trial. The study aims to evaluate the safety and preliminary efficacy of intravenous EVs derived from human iPSCs in acute ischemic stroke. In the initial phase, participants will be administered escalating doses of 20 μg/kg, 40 μg/kg, and 80 μg/kg from human-iPSCs-derived EVs. The second phase involves an expanded safety assessment, where 20 subjects will be randomly assigned to receive either the EVs treatment or a placebo. The dose level will be determined by the Data Safety Monitoring Board based on findings from the initial phase.

### Safety of Extracellular Vesicles in Clinical Trials

Although phase I and II trials are designed to establish the safety and potential toxicity of a treatment, the side effects of EV-based therapies have not been extensively addressed. This is because these therapies are relatively new, and many clinical trials are still ongoing. Additionally, phase I trials are pilot studies with a very small patient population. Moreover, the primary focus of the trials reported in Table 5 is on assessing the efficacy of the therapies, rather than conducting a thorough evaluation of their toxicity. Indeed, it has been reported that, in general, the analysis of adverse effects in clinical trials is often inconsistent or underreported [138]. 

Despite these considerations, it is important to note that some of the ongoing studies (Table 5) involve allogeneic sources, meaning the EVs are derived from a different individual of the same species. Consequently, in terms of safety, this approach may carry a higher risk of immune rejection or adverse immune responses due to genetic differences between the donor and recipient.

However, a recent meta-analysis on the safety of EV-based therapies in clinical trials found that despite the high heterogeneity of the studies, there was a higher incidence of adverse events following the administration of autologous treatments compared to allogeneic treatments [139]. The study reported that out of the 335 patients evaluated across various trials, 5 experienced serious adverse effects, including liver dysfunction, pyrexia, vomiting, and acute asthmatic exacerbation. Additionally, some adverse events were localized to the injection site, such as redness and pain, though these reactions were only reported in two of the studies. The results also suggest that engineering EVs does not lead to a higher incidence of severe adverse events compared to non-engineered EVs. It is also important to emphasize that not only the content but also the different membrane components of EVs could play a role in numerous processes related to the safety of these treatments. For example, different studies point to their involvement in events such as hemostasis and thrombosis (for a review on this topic, see [140]). 

Among the seven clinical trials selected in Table 5, only two have published data. The first, NCT04388982 [136] on Alzheimer’s disease patients, and the second, NCT03384433 on stroke patients, reported no adverse effects [137].

Adverse events and severe adverse events are expected to be reported according to the respective clinical trial pages on ClinicalTrials.gov (last check on 6 of September 2024): NCT04202783 (from baseline to 24 weeks); NCT05490173 (during the first week); NCT05886205 (assessed at 24 weeks post-administration); NCT06138210 (assessed at 14 and 90 days for severe adverse events from the trial’s start). No information is currently provided regarding the collection of safety data for the following clinical trials: NCT04202770 and NCT04202783.

## 4. Conclusions

In summary, the studies mentioned above demonstrated the potential of EVs as biomarkers for CNS diseases; employing EVs as molecular diagnostic markers showed promising applications. Nevertheless, further studies are necessary to improve the effectiveness of EV-based biomarkers for clinical diagnosis and disease progression of AD, PD, and ALS. Several issues need to be addressed. For instance, the low levels of pathogenic proteins or nucleic acids that cross the blood–brain barrier and reach the peripheral blood can be influenced by multiple organs. Thus, plasma brain-derived EVs may not sensitively reflect minor pathological changes in the brain. Combining multiple biomarkers within EVs may enhance the accuracy and sensitivity of CNS diseases detection, enabling early intervention and improved patient outcomes. Although the clinical use of EVs is still in its early stages, there has been a surge of interest in clinical trials involving EVs across various pathologies. Further clinical trials are ongoing to validate the utility of these EV cargo components as reliable biomarkers and as nanocarriers of therapeutic molecules derived from stem cells. Stem-cell-derived EVs have shown great potential to offer therapeutic advantages. An essential question is whether EVs originating from different types of stem cells demonstrate varied protective or therapeutic effects in pathological environments. There are unresolved questions concerning the purity of different EVs subtypes, individual responses to chronic administration, EV quantity, and other aspects of therapeutic protocols. Indeed, a limitation of this review and the reviewed studies aligns with the common challenges faced in studies on EVs. Due to variability in extraction quality and insufficient characterization of EVs, it is not always easy to assess the quality of individual studies. In fact, after extraction, it is common to find other non-vesicular nanoparticles (such as albumin, exomeres, supermeres, vaults, and viral components) that could potentially influence both the identification of biomarkers and the evaluation of the therapeutic effects in EVs preparations. Recent findings indicate that nucleic acid components are found mostly in NVEPs compared to vesicular particles [141,142]. However, an open question remains regarding the extent to which these vesicles contribute to the transfer of this genetic material to other cells, as well as the mechanisms by which this occurs [30]. Further experiments are needed to determine whether the role of biomarkers or therapeutic functions can be specifically attributed to non-vesicular nanoparticles or EVs conclusively. Furthermore, for the clinical implementation of stem-cell-derived EVs, it is crucial to establish standardized purification protocols to prevent the inadvertent transfer of genetic and/or protein components and to mitigate the risk of immune system activation.

## Figures and Tables

**Table 1:** Summary of potential biomarkers in EV-containing preparations for Alzheimer’s disease and their characteristics.

Biomarker	Source EVs	Isolation Technique	Characterization Technique	Function	Reference
Aβ42, t-tau (total), p-T181-tau, p-S396-tau	BDE from Plasma/Serum	ExoQuick Exosome isolation kit Chromatography using qEV columns	ELISA SIMOA Luminex	Significantly different in patients with AD compared to controls, regardless of the stage of the disease	[37]
P-S396-tau, P-T181-tau, Aβ1–42	nEVs from Plasma	ExoQuick/ Immunoabsorption	NTA ELISA	To predict the development of AD up to 10 years before clinical onset	[41,42]
P-T181-tau, P-S396-tau, Aβ1-42, NRGN, REST	nEVs from Plasma	ExoQuick/ Immunoabsorption	TEM NTA ELISA	To predict conversion of MCI to AD dementia	[41,42]
Aβ42, Aβ42/Aβ40 ratio, t-tau, ratio p-T181-tau/tau, miR-384	nEVs from Plasma	ExoQuick/ Immunoabsorption	TEM Western blot Laser scattering microscopy ELISA	Early detection and risk assessment of aMCI/AD	[48]
p-T181-tau	CSF	Ultracentifugation/Sucrose gradient	Immunoblot Electron Microscopy ELISA Mass Spectrometry	Increased in early AD	[39,40]
p-T181-tau and t-tau/Aβ42 ratio	Plasma	Total Exosome Isolation reagent/Filtrations and precipitations	TEM Western blot NTA Luminex	High accuracy to define MCI and AD staging	[46]
Aβ42/40 ratio	Plasma and CSF	Ultracentrifugation	Fluorescent probe PKH26 Immunofluorescence TEM NTA Immunoblot	Significantly higher in EVs isolated from patients with AD CSF compared with EVs from control subjects CSF	[46,49]
tau, p-T181-tau, p-tau-231, insulin signaling biomarkers	nEVs from Serum	Precipitating solution/Immunoabsorption	NTA ELISA	Higher levels in cognitive decline less severe than MCI or dementia	[50]
Aβ1-42, tau, p-T181-tau, p-S396-tau, REST, NRGN, cathepsin D	nEVs from Plasma	ExoQuick/ Immunoabsorption	ELISA	A different tendency is observed in AD compared to controls	[42,51]
P-S396-tau, Aβ1–42	Plasma	ExoQuick	TEM NTA ELISA	Significantly higher in AD compared to healthy controls	[52,53]
Aβ42, T-tau, P-T181-tau	nEVs from Plasma, CSF	ExoQuick/ Immunoabsorption	TEM Western blot ELISA	Levels were higher in AD group than in aMCI and control groups; levels in MCI and AD were strongly correlated with CSF levels	[52,53]
Aβ1-42	nEVs from Plasma	ExoQuick/ Immunoabsorption	TEM NTA Western blot ELISA	In combination with a test assessing olfactory function, it improved prediction of the conversion from MCI to AD	[54]
AACT C4BPα	Serum	ExoQuick/ Column-based ExoSpin Blood Exosome Purification Kit	TEM NTA Western blot Mass Spectrometry	Elevated levels of AACT and reduced levels of C4BPα in AD diagnosis	[55]
Gelsolin	Serum	Precipitation and column-based method	NTA TEM Western blot ELISA	Lower in dementia	[56]
BACE1-AS	Serum	ExoQuick	TEM NTA Western blot	Elevated in AD	[57]
BACE-1 sAPPβ GDNF	Astrocyte-derived EVs from plasma	ExoQuick/ Immunoabsorption	NTA ELISA	Levels of BACE-1 and sAPPβ were significantly higher, while levels of GDNF were significantly lower in patients with AD compared to controls	[58]
tau	EVs derived from microglia	Centrifugation/ Sucrose density gradient/Immunoprecipitation	TEM Tunable Resistive Pulse Sensing Analysis Immunoblotting	Increased levels of tau were detected in patients with AD compared to controls	[59]
NfL	Plasma	Exosome isolation kit	TEM Western blot SIMOA	Higher levels in patients with AD	[61]
Ten proteins (distinct immunoglobulins, fibronectin, and apolipopro-teins)	Plasma	-	-	Biomarkers of sporadic AD	[62]
Lipids	Brain-derived EV from plasma	Exoquick-LP/ Immunoprecipitation	NTA UPLC-MSMS	Lipid composition differences between AD and control individuals	[63]
miR-135a miR-384 miR-193b	Serum	Exosome Isolation kit	NTA Western blot qRT-PCR	miR-135a and miR-384 were up-regulated while miR-193b was down-regulated in patients with AD compared with controls	[64]
miRNAs-125b-3p miRNA-451a	Serum	Exoquick	TEM NTA Western blot Sequencing (Illumina HiSeqTM) qRT-PCR	Differentiates the AD group from the healthy group	[65]
miR-483-5p miR-502-5p	Plasma	Ultracentrifugation	TEM Western blot qRT-PCR	High levels in elderly people with MCI with respect to control group	[66]
Aβ1–42, P-S396-tau	Urine	Exoquick	TEM NTA ELISA	Higher levels in AD than in healthy controls	[67]
Aβ oligomer/fibril, Aβ, and P-tau	Saliva	PEG precipitation	TEM NTA Western blot	Increased levels in AD and cognitive impaired patients compared to controls	[68]
miRNA-485-3p	Saliva	ExoRNeasy Midi Kit	TEM Fluorescent probe PKH67 Western blot qRT-PCR	Levels positively correlated with Aβ deposition in the brain and high predictive value for Aβ-PET positivity	[69]

AACT = alpha-1-antichymotrypsin; aMCI = amnesic mild cognitive impairment; BACE-1 = β-site amyloid precursor protein-cleaving enzyme 1; BACE1-AS = BACE1-antisense transcript; BDE = brain derived exosomes; C4BPα = C4b-binding protein alpha chain; CSF = cerebrospinal fluid; GDNF = glial-derived neurotrophic factor; MCI = mild cognitive impairment; nEVs = neuronal extracellular vesicles; NfL = neurofilament light chain; NRGN = neurogranin; NTA = nanoparticle tracking analysis; PEG = polyethylene glycol; p-S396-tau = tau phosphorylated at serine 396; p-T181-tau = tau phosphorylated at threonine 181; REST = repressor element 1-silencing transcription factor; sAPPβ = β soluble amyloid precursor protein; TEM = transmission electron microscopy; UPLC-MSMS = ultra-performance liquid chromatography–tandem mass spectrometry.

**Table 2:** Summary of potential biomarkers in EV-containing preparations for Parkinson’s disease and their characteristics.

Biomarker	Source EVs	Isolation Technique	Characterization Technique	Function	Reference
SNAP-25, GAP-43, synaptotagmin-1	Plasma	exoEasy Maxi Kit	TEM NTA Western blot	To predict deterioration of motor function, particularly postural instability and gait disturbance symptoms	[75]
α-synuclein, tau, and Aβ 1-42	Plasma	exoEasy Maxi kit	TEM NTA Western blot	Predictive of motor and cognitive dysfunction progression at the 1-year follow-up	[76]
IL-1β and IL-6	Plasma	exoEasy Maxi Kit	TEM NTA Western blot	Predictors of the progression of postural instability and gait disturbance	[77]
α-synuclein	nEVs from Serum	EV precipitation reagent/ Immunocapture	- syn SAA	Higher in early and advanced forms of the disease compared to healthy individuals; in combination with prodromal markers, it should be considered in the stratification of those at high risk of developing PD and related Lewy body diseases	[70,78,79]
α-synuclein Clusterin	nEVs from Serum	EV precipitation reagent/ Immunocapture	NTA TEM Western blot Mass spectrometry	Prediction and differentiation of PD from atypical Parkinsonism	[80,81]
α-synuclein, p-Tyr-181-tau, phosphorylated insulin receptor substrate-1	nEVs from Serum	Exoquick/ Immunoprecipitation	Cryogenic TEM NTA Western blot Immunofluorescence SIMOA	Cognitive prognosis in PD	[83]
α-synuclein	Astrocyte-derived EVs from Plasma	Ultracentrifugation/Immunoprecipitation	NTA TEM Western blot Flow cytometry Multiplexed immunoassays	Higher levels were found in the PD group compared to healthy controls	[84]
RALB, POR, SMPD3, MAPT, DDX6, ILK, OGN, CTSS, PPP1CC, FLII, IGKV1-8, SHMT1, RPL35A	CSF	SmartSEC column HT kit	ExoView Tetraspanin kit NTA NanoOrange Protein Quantitation Kit Mass spectrometry SIMOA	Candidates that differentiated healthy controls from PD	[93]
miR-44438	Plasma	Molecular beacon-based assay	TEM NTA Flow cytometry	Early detection and progress monitoring of α-synucleinopathies	[95]
miR-128	Plasma	miRCURY Exosome Plasma kit	NTA qRT-PCR	Decreased in PD; associated with neural degeneration and 6-OHDA-mediated neuronal apoptosis	[97]
29 small RNAs	nEVs from Serum	Exoquick/ Immunoprecipitation	TEM NTA RNA-seq	Expressed differentially between patients with PD and controls	[98]
miR-7-5p, miR-4665-5p, miR-5001-3p, and miR-550b-3p	Plasma	exoRNeasy Midi kit	TEM NTA Western blot EV-RNA size (Bioanalyzer) High throughput sequencing	Patients with REM sleep behavior disorder and higher levels of these miRNAs are more likely to develop PD	[99]
miR-153, miR-409-3p, miR-10a-5p, let-7g-3p, miR-1, and miR-19b-3p	CSF	Ultracentrifugation/Sucrose gradient	Flow cytometry TEM miRNeasy Serum Kit EV-RNA size (Bioanalyzer) TaqMan Array Human MicroRNA RT-qPCR	Distinguish PD from both healthy individuals and those with other neurodegenerative conditions like AD	[100]
α-syn_Olig_, α-syn_Olig_/α-synTotal ratio	Saliva	XYCQ EV t KIT/Immunoprecipitation	TEM NTA Western blot Electrochemiluminescence immunoassays	To differentiate PD from healthy control with high sensitivity and specificity	[101,102,103,104,105]
Calbindin, SNAP23	Urine	Ultracentrifugation	NTA TEM Western blot Mass spectrometry	Increased levels in PD respect to control	[101,102,103,104,105]
DJ-1	Urine	Microfiltration/Centrifugation	Western blot	Significantly higher in male patients with PD than in male non-PD controls and increased in an age-dependent manner in male patients with PD	[101,102,103,104,105]
Ser(P)-1292 LRRK2	Urine	Ultracentrifugation	Cryo-electron micrographs Western blot	Elevated in idiopathic PD and correlated with the severity of cognitive impairment and difficultly in accomplishing the activities of daily living	[101,102,103,104,105]
Phosphorylation of Rab10	Urine	Ultracentrifugation	Western blot	Higher levels are associated with worse disease progression	[107]
Rab8, phosphorylation at pS910-LRRK2 and pS935-LRRK2	Urine	Ultracentrifugation	NTA TEM Western blot	PD was associated with increased Rab8 levels and decreased pS910-LRRK2 and pS935-LRRK2	[108]

CSF = cerebrospinal fluid; GAP-43 = growth-associated protein 43; LRRK2 = leucine-rich repeat kinase 2; nEVs = neuronal extracellular vesicles; NTA = nanoparticle tracking analysis; pS910-LRRK2 = LRRK2 phosphorylated at serine 910; pS935-LRRK2 = LRRK2 phosphorylated at serine 935; p-Tyr-181-tau = tau phosphorylated at tyrosine 396; SAA = seed amplification assay; SNAP-25 = synaptosome-associated protein 25; TEM = transmission electron microscopy.

**Table 3:** Summary of potential biomarkers in EV-containing preparations for amyotrophic lateral sclerosis and their characteristics.

Biomarker	Source EVs	Isolation Technique	Characterization Technique	Function	Reference
Coronin-1a	Plasma	Exosome isolation kit	TEM NTA MS/MS	Increased in patients with ALS compared to controls	[109]
SOD1	Plasma	Ultracentrifugation	TEM NTA Western blot	Increased in patients with ALS compared to controls	[112]
TDP-43	Serum	Glycan-recognition base magnetic bead (EXOBead)	Flow cytometry	Increased at the 3-month and 6-month follow-up periods	[113]
NIR	CSF	Size exclusion chromatography	TEM ELISA Western blot LC–MS/MS	Increased levels in patients with ALS	[119]
miR-146a-5p	CSF	Ultracentrifugation	Tunable Resistive Pulse Sensing RT-qPCR	Expressed in patients with ALS	[120]
miR-15a-5p and miR-193a-5p	Plasma	Precipitation with Vn96 peptide	NGS	Diagnostic potential of ALS and association with disability progression	[121]
30 miRNAs	nEVs from Plasma	Polyethylene glycol precipitation (ExoQuick)/ Immunoprecipitation	NTA Flow cytometry Microarray	Differential regulation in ALS compared to healthy control	[122]
miR-146a-5p, miR-199a-3p, miR-151a-3p, miR-151a-5p, and miR-199a-5p	nEVs from Plasma nEVs from Plasma	ExoQuick/ Immunoprecipitation ExoQuick/ Immunoprecipitation	NTA ELISA Exo-Check exosome antibody array NGS RT-qPCR	Up-regulated in patients with ALS compared to healthy controls	[123,124]
miR-4454, miR-10b-5p, and miR-29b-3p				Down-regulated in ALS compared to healthy controls	

ALS = amyotrophic lateral sclerosis; CSF = cerebrospinal fluid; MS/MS = tandem mass spectrometry; nEVs = neuronal extracellular vesicles; NGS = next-generation sequencing; NIR = INHAT repressor; SOD1 = superoxide dismutase; TDP-43 = TAR DNA-binding protein-43.

**Table 4:** Clinical trials using EVs as biomarker for CNS diseases. Source: ClinicalTrials.gov.

Clinical Trial Number	Conditions	Title	Year of Initiation	Source EVs	Application	Country	Status
NCT03381482	Alzheimer’s disease Age: 40 years to 85 years (adult, older adult)	Ectosomes, New Biomarkers of tau Pathology?	2017	CSF and blood sample	To decipher the nature of tau secreted in plasma and cerebrospinal fluids collected from healthy controls and patients with AD and to determine if the presence of tau inside vesicles is influenced by the pathology	France	Recruiting; estimated study completion date: December 2023
NCT04121208	Alzheimer’s disease/mild cognitive impairment Age: 50 years to 99 years (adult, older adult)	MIcroglial Colony Stimulating Factor-1 Receptor (CSF1R) in Alzheimer’s Disease (MICAD)	2019	CSF sample	To assess EVs as indicators of altered microglial cell activity in the brain	United Kingdom	Study completion date: February 2022
NCT05163626	Alzheimer’s disease Age: 50 years to 80 years (adult, older adult)	Combined Aerobic Exercise and Cognitive Training in Seniors at Increased Risk for Alzheimer’s Disease	2021	Neuronal EVs from blood sample	To assess the effects of a long-term combined aerobic exercise and cognitive training program on cognitive function and the predictive biomarkers (blood neuro-exosomal synaptic proteins: GAP43, neurogranin, SNAP25, and synaptotagmin 1) in seniors at increased risk of AD with abnormally decreased levels of the biomarkers To determine the relationship of biomarker changes with cognitive function in these people; to confirm the predictive value of the blood neuroexosomal synaptic proteins for AD in a longitudinal setting	China	Not yet recruiting; estimated study completion date: December 2034
NCT03275363	Neurocognitive disorder; Mild cognitive impairment; Alzheimer’s dementia; vascular dementia; Age-related cognitive decline Age: 65 years and older (older adult)	The University of Hong Kong Neurocognitive Disorder Cohort	2017	Blood sample	To study biomarkers that predict cognitive and functional decline	China	Recruiting status unknow; estimated study completion date: April 2022
NCT01811381	Mild cognitive impairment. Age: 50 years to 90 years (adult, older adult)	Curcumin and Yoga Therapy for Those at Risk for Alzheimer’s Disease	2014	Blood sample	To assess if the combination of curcumin and aerobic exercise significantly changes the levels of biomarkers (such as RNAseq on exosomes) thought to be associated with MCI (blood biomarker levels among those receiving supplements vs. placebo)	EEUU	Estimated study completion date: December 2020
NCT01681602	Alzheimer’s disease Age: 50 years to 90 years (adult, older adult)	Effect of Physical Exercise in Alzheimer Patients (ADEX)	2012	Blood sample	To assess the effect of physical exercise in patients with AD and analyze changes in the content of neuron-derived EVs	Denmark	Study completion date: June 2014
NCT03852901	Adults at least 55 years old without diabetes Age: 55 years and older (adult, older adult)	Sodium-glucose Co Transporter 2 (sGLT2) Inhibitor and Endogenous Ketone Production	2019	Blood sample	To study how taking empagliflozin affects systemic and brain metabolism, including ketone levels in people without diabetes; to analyze the expression of receptors and mediators of ketone metabolism in plasma exosomes and IGF-1/insulin cascades in exosomes from subjects taking a sGLT2 inhibitor	EEUU	Study completion date: December 2021
NCT01860118	Parkinson’s disease Age: 21 years to 99 years (adult, older adult)	LRRK2 and Other Novel Exosome Proteins in Parkinson’s Disease	2013	Blood samples and urine	To identify biomarkers associated with PD susceptibility and/or progression in exosome-proteomes derived from patients with PD versus controls; to determine if LRRK2 expression and/or phosphorylation are significantly lowered in the exosomes of individuals treated with the potent LRRK2 kinase inhibitor sunitinib (a multi-kinase inhibitor compound); to establish an assay for on-target effects for future LRRK2 inhibitor clinical trials	EEUU	Study completion date: May 2018
NCT05452655	Parkinson’s disease Age: 50 years to 85 years (adult, older adult)	Intensive Multidisciplinary Rehabilitation and Biomarkers in Parkinson’s Disease	2020	Blood sample	To identify accessible and measurable EVs as biomarkers for monitoring the effects induced by an intensive motor and cognitive rehabilitation program and to predict patient responsiveness	Italy	Estimated study completion date: December 2023
NCT05320250	Parkinson’s disease Age: 50 years to 85 years (adult, older adult)	Saliva and Extracellular Vesicles for Parkinson’s Disease	2021	Saliva sample	The objective of this project is the validation of Raman analysis of saliva and salivary extracellular vesicles (EV) for the differential diagnosis of Parkinson’s disease (PD) and atypical Parkinsonism; the proposed diagnostic method can be integrated in the preliminary assessment and monitoring of the patient by providing a quickly and repeatable measurable biomarker; in the end, this will lead to the personalization of the rehabilitation path and provide an indication on the outcome of the rehabilitation treatment	Italy	Recruiting; estimated study completion date: December 2024
NCT05902065	Parkinson’s disease Age: 18 years and older (adult, older adult)	Effect of a Progressive Treadmill Training Protocol for Parkinson’s Disease	2022	Blood sample	To identify predictive and indicative biomarkers of an outcome measure of rehabilitation using extracellular vesicles assessed via Raman spectroscopy	Italy	Recruiting; estimated study completion date: December 2023
NCT03775447	Parkinson disease Age: 18 years or older at time of PD diagnosis	Fox BioNet Project: ECV-003	2019	CSF sample	To optimize pre-analytical cerebrospinal fluid (CSF) extracellular vesicle isolation protocols for increasing the detection of LRRK2 activity in human CSF	EEUU Canada	Recruitment status: completed; study completion date: December 2019
NCT04603326	Parkinson’s disease Age: 30 years and older (adult, older adult)	FoxBioNet: ECV (Extracellular Vesicle) 004	2021	CSF sample	To identify reliable markers of LRRK2 activity; to assess the ability of the assay or a combination of assays to differentiate pathogenic LRRK2 variant manifesting and non-manifesting carriers, idiopathic PD, and healthy non-carriers	EEUU Canada	Recruitment status: completed; study completion date: July 2023
NCT05279599	Traumatic brain injury Age: 18 years to 80 Years (adult, older adult)	Application of Circulating Extracellular Vesicles in Early Disease Assessment and Prognosis After Traumatic Brain Injury	2022	Blood sample	The purpose of this study was to observe the relationship between the changes of circulating extracellular vesicles and disease development and outcome in patients with traumatic brain injury and to find early serum markers and potential intervention targets for disease monitoring in patients with traumatic brain injury	China	Recruiting status: unknow; estimated study completion date: November 2022
NCT05370105	Stroke Age: 35 years to 75 years (adult, older adult)	Extracellular Vesicles as Stroke Biomarkers (EXO4STROKE)	2018	Blood sample	Characterization of circulating extracellular vesicles/exosomes as new predictive biomarkers in stroke patients; the study aimed to demonstrate the ability of the surface plasmon resonance imaging (SPRi) biosensor to revealing differences in the relative amount of specific cell-derived EV subpopulations and in their cargo during disease progression and rehabilitation; induced recovery; the study further aimed to provide support for using the proposed SPRi-based biosensor for the detection and characterization of circulating EVs in order to evaluate the efficacy of rehabilitation protocols and regenerative therapies and to identify new biomarkers for the profiling of stroke patients to personalize the rehabilitation therapies	Italy	Active (not recruiting)
NCT06319742	Stroke Age: 18 years and older (adult, older adult)	Extracellular Vesicle Surface Markers In Acute Cerebrovascular Syndromes. (ElViS-ACS)	2022	Blood sample	To determine the performance of EV profiling (analysis of antigens expressed on vesicles surface) added to a structured clinical (PREDISC score) and imaging evaluation (brain MRI); to discriminate transient ischemic attacks (TIAs) from TIA mimics (non-ischemic events); to determine the performance of EV profiling to differentiate between TIAs (with negative MRI for acute lesion) and acute ischemic strokes (MRI confirmed); to determine the performance of EVs profiling to categorize pre-specified causes of ischemic events	Italy	Recruiting; estimated study completion: January 2026

**Table 5:** Clinical trials using EVs therapy for CNS diseases. Source: clinicaltrials.gov.

Clinical Trial Number	EVs Source	Intervention/Treatment	Conditions	Phase	Country	Status
NCT03384433	Allogenic MSC-derived EVs enriched with miR-124	Stereotaxis/Intraparanchymal (one month after attack)	Acute ischemic stroke Age: 40 years to 80 years (adult, older Adult)	1/2	Iran	Estimated study completion date: 17 December 2021
NCT04202770	EVs derived from healthy, full-term Cesarean section amniotic fluid	Transcranial-focused ultrasound as a means of enhancing delivery of intravenous EVs to the subgenual target for patients with refractory depression, the amygdala for patients with anxiety, and the hippocampus for patients with cognitive impairment due to neurodegenerative disease	Depression, anxiety, dementia Age: 18 years and older (adult, older adult)	1	USA	Recruitment suspended (pending COVID-19 pandemic; pending status of product development); estimated study completion date: December 2024
NCT04202783	EVs carrying neonatal stem cell products	Patients will be given 3 mL of the EVs product intravenously, which contains about 45 mg of the EVs product containing 15–21 million neonatal stem cell products, and 3 mL of the EVs hyperconcentrate product delivered epineurally using ultrasound guidance, which contains about 15 mg of the EVs product carrying 5–7 million neonatal stem cell products	Craniofacial neuralgia Age: 18 years and older (adult, older adult)	1	USA	Recruitment suspended (pending COVID-19 pandemic; pending product development); estimated study completion date: December 2024
NCT04388982	Allogenic adipose MSC-derived EVs	Administrated for nasal drip; three doses of MSCs-Exos: Low dosage (5 μg); Mild dosage (10 μg); High dosage (20 μg); Total volume: 1 mL; Frequency: twice a week; Duration: 12 weeks	Alzheimer’s disease (mild-to-moderate dementia) Age: 50 years and older (adult, older adult)	1/2	China	Estimated study completion date: August 2022
NCT05490173	MSC-derived EVs	Intranasal administration; surviving extremely low birth weight (ELBW) infants will be randomized to receive (group 1) and not receive EVs (control group): Group 1—neonates will receive EVs (one dose will be obtained from a daily conditioned culture medium of 120 million MSCs) suspended in 500 µL of phosphate buffer in each nostril at 50 µL with an interval of 2–3 min; the therapeutic course will consist of five instillations with an interval of 1 days.	Neurodevelopmental disorders of prematurity; premature newborns of gestational age 25/0–27/6 weeks Ages: 1 day to 3 days (child)	1	Russia	Not yet recruiting; estimated study completion date: December, 2026
NCT05886205	iPSC-derived EVs	Administrated for nasal drip for 12 weeks. Group1 (low-dose group)—eight patients are treated with 2 μg iPSC-EVs in 200 μL; Group2 (mid-dose group)—eight patients are treated with 6 μg iPSC-EVs in 200 μL; Group3 (mid-dose group)—eight patients are treated with 18 μg iPSC-EVs in 200 μL; Group4 (dose expansion)—ten patients are treated with iPSC-EVs in 200 μL	Refractory focal epilepsy Age: 18 years to 70 years (adult, older adult)	Early 1	China	Recruiting; estimated study completion date: November, 2025
NCT06138210	Human-iPSC-derived EVs	Intravenous injection once a day for 7 days In part 1: cohort 1 receive 20 μg/kg, cohort 2 40 μg/kg, and cohort 3 80 μg/kg; if no dose-limiting toxicities (DLTs) are observed for 2 weeks after administration of the first injection, a new cohort will be enrolled at the next planned dose level; if DLTs are observed in one participant in the cohort, another three participants will be treated at the same dose level; dose escalation will be stopped until DLTs are observed in >33% of the participants In part 2, 20 subjects will be randomized in a 1:1 ratio [EVs (*n* = 10) or EVs placebo (*n* = 10)]; the dose level will be determined by the Data Safety Monitoring Board based on part 1.	Acute ischemic stroke Age: 18–70 years	1	China	Not yet recruiting; estimated study completion date: August, 2025

COVID-19 = coronavirus disease 2019; EVs = extracellular vesicles; MSC = mesenchymal stromal cell; iPSC = induced pluripotent stem cell; NCT = national clinical trial.

## Data Availability

Not applicable.
